# Elimination of a signal sequence-uncleaved form of defective HLA protein through BAG6

**DOI:** 10.1038/s41598-017-14975-9

**Published:** 2017-11-06

**Authors:** Koki Yamamoto, Mizuki Hayashishita, Setsuya Minami, Kanji Suzuki, Takumi Hagiwara, Aya Noguchi, Hiroyuki Kawahara

**Affiliations:** Laboratory of Cell Biology and Biochemistry, Department of Biological Sciences, Tokyo Metropolitan University, Tokyo, 192-0397 Japan

## Abstract

A portion of newly synthesized transmembrane domain proteins tend to fail to assemble correctly in the lumen of the endoplasmic reticulum, thus resulting in the production of a signal sequence-uncleaved form of the defective species. Although the efficient degradation of these mistargeted polypeptides is crucial, the molecular mechanism of their elimination pathway has not been adequately characterized. In this study, we focused on one such cryptic portion of a defective transmembrane domain protein, HLA-A, and show that a part of HLA-A is produced as a signal sequence-uncleaved labile species that is immediately targeted to the degradation pathway. We found that both BAG6 and proteasomes are indispensable for elimination of mislocalized HLA-A species. Furthermore, defective HLA-A is subjected to BAG6-dependent solubilization in the cytoplasm. These observations suggest that BAG6 acts as a critical factor for proteasome-mediated degradation of mislocalized HLA-A with a non-cleaved signal sequence at its N-terminus.

## Introduction

Mislocalized proteins (MLP) are type of defective newly synthesized polypeptides that fail to assemble correctly in their target organelles^1–5^. Emerging evidence suggests that a non-negligible proportion of newly synthesized endoplasmic reticulum (ER) proteins fail to assemble correctly in the ER membrane^1,6,7^. Indeed, a surprisingly low efficiency of ER targeting for some membrane proteins has been reported, including one case with mammalian GPI-anchored glycoprotein prion (PrP), which resulted in the production of cytosolic mislocalized species^1,2,8^. The efficient removal of these defective polypeptides is important because the accumulation of such mislocalized membrane proteins can result in their aggregation in the cytoplasm, promiscuous interactions with vital cytosolic proteins, and eventually cause neurodegeneration, diabetes, and immune disorders^8–12^. Thus, the recognition and removal of mislocalized proteins is of significance to avoid such potential proteotoxic stress. Nevertheless, the molecular mechanism of the elimination pathway that targets defective species, especially for transmembrane domain (TMD) proteins, before they reach the lumen of the ER has only recently received increased attention.

Accumulating evidence suggests that the substrates for both the co- and post-translational ER targeting pathways are polyubiquitinated in a BAG6 (also known as BAT3 or Scythe)-dependent manner when their targeting fails^2–4,13–17^. As an example, PrP displayed preferential stabilization as a non-glycosylated precursor species when BAG6 was knocked down^2^. We also recently reported that the signal sequence (SS)-truncated (and thus artificially mislocalized) single-pass TM protein IL-2Rα is captured by the UBQLN4/BAG6 complex in an unembedded TMD-dependent manner^4^. BAG6 is a hydrophobicity-oriented chaperone/holdase^18–24^ that has been shown to be a receptor for tail-anchored (TA) protein biogenesis^25–31^ and for targeted degradation of newly synthesized defective polypeptides with exposed hydrophobicity in the cytosol^2,7,19,20,32–34^. However, the critical functions of BAG6 in the elimination of the SS-uncleaved form of mislocalized TMD proteins have not been adequately proven and further experimental verification is awaited.

In this study, we focused on the cryptic portion of a defective TMD protein, which is difficult to detect, partly because it appears to be degraded immediately upon synthesis in the cytosol and partly because of the limited available methodologies for distinguishing defective species from their successfully assembled counterparts. With T7-tagging upstream of an SS, we can specifically discriminate mislocalized species of defective TMD polypeptides that fail to be processed at the N-terminal SS by ER luminal signal peptidase. Using this mislocalized TMD protein model, we attempted to uncover the machinery underlying the targeted elimination of such defective species. Here, we provide evidence that BAG6 and proteasomes act as critical factors for the elimination of the SS-uncleaved (and therefore non-incorporated into the ER lumen) form of the TMD polypeptide, human leukocyte antigen (HLA-A).

## Results

### Detection of the signal sequence-uncleaved form of defective HLA-A

**Figure 1:**
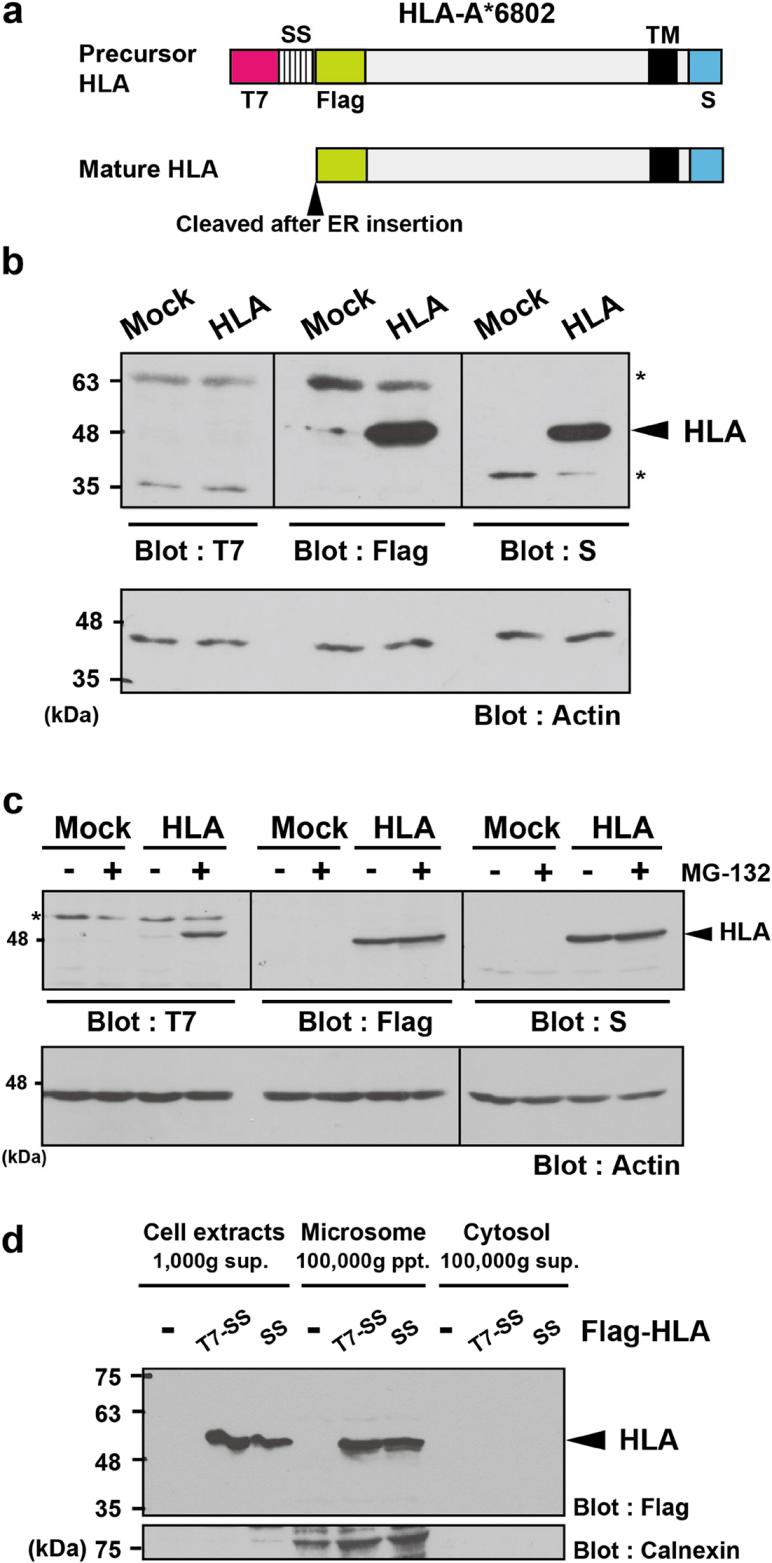
Detection of the SS-uncleaved form of HLA-A. (**a**) Schematic representation of the HLA-A*6802 proteins examined in this experiment. The T7‐tag in the precursor form of HLA-A*6802 is located upstream (N-terminus) of the signal sequence (SS), thus, SS cleavage by signal peptidase occurring immediately after ER luminal incorporation would result in the loss of the T7 signal, while the Flag- and S-tag signals are detectable even in the mature form of HLA-A*6802. (**b**,**c**) HeLa cells transfected with the HLA-A*6802 expression vector were treated with or without 10 μM MG-132. At 4 h after MG-132 treatment, the cells were lysed and the accumulation of HLA-A*6802 proteins was examined by either anti-T7, anti-Flag, or anti-S-tag antibodies. These electrophoresis samples were derived from the same experiment and the blots were processed in parallel. Arrowheads indicate the HLA-A*6802 signals. Mock indicates empty vector transfection. Actin was used as a loading control. The band indicated by an asterisk is non-specific. **(d)** HeLa cells were transfected with expression vectors encoding T7-tagged HLA or the N-terminally non-tagged HLA (both of which possess a Flag-tag following their SS). After preparation of the microsomal fractions, these were blotted with the anti-Flag antibody to detect incorporation of HLA-A*6802 into microsome. Note that the cytosolic fractions (100,000 x*g* sup.) barely contain any Flag antigens, also suggesting that the majority of HLA was incorporated into the microsomal fraction. Full-length images of blots are presented in Supplementary Fig. S1.

Successful biogenesis of TMD proteins is mostly mediated through SS recognition by the signal recognition particle (SRP), and these complexes are recruited to the surface of the rough ER, where newly synthesized polypeptides are assembled in the ER membrane^35–45^. The HeLa cell-endogenous major histocompatibility complex (MHC)-class I molecule, HLA-A*6802, is a single-pass TM protein that contains a typical SS for ER insertion at its N-terminus and a TMD at its C-terminus (Fig. 1a). It has generally been believed that most single-pass TM proteins are integrated efficiently into the ER membrane in an SS-mediated co-translational process. To estimate whether this process is indeed accomplished efficiently within HeLa cells, we explored the possible detection of mistargeted HLA-A species.

As the SS for ER targeting is immediately cleaved off by ER luminal signal peptidase^46^, and SS peptides are subsequently discarded after their successful co-translational insertion into the ER lumen, it might be possible to discriminate mistargeted (and thus SS-uncleaved) HLA-A species by tagging an antigenic sequence just upstream of an SS (Fig. 1a). To this end, we have established an expression vector encoding N-terminally T7-tagged (upstream of an SS), Flag-tagged (downstream of the cleavage site for the ER signal peptidase), and C-terminally S-tagged HLA-A*6802 protein (Fig. 1a).

As anticipated, anti-S-tag- as well as anti-Flag-tag-immunoblot experiments clearly showed the stable expression of HLA-A*6802 protein in HeLa cells (Fig. 1b). In contrast, the anti-T7-tag immunosignal could barely be detected in identical cell lysates (Fig. 1b), superficially implying that SS removal at the ER lumen (and thus successful assembly in the ER) was executed very efficiently (note that our detection sensitivities with the anti-Flag and anti-T7 tags were adjusted equivalently in Fig. 1b). However, we subsequently noticed that addition of the protease inhibitor MG-132 (Z-Leu-Leu-Leu-CHO) to the cell culture medium resulted in substantial accumulation of a T7-specific signal at 50 kDa, a molecular weight equivalent to that of the unprocessed HLA-A*6802 precursor, compared with the case for the DMSO solvent control (Fig. 1c, T7-blot). It should be noted that MG-132 treatment did not affect the intensity of the Flag- and S-tag positive total HLA-A signals (Fig. 1c, Flag- and S-blots), suggesting that successfully ER-assembled (and therefore mature) HLA-A*6802 is a stable product. These observations suggest that at least a proportion of ectopically expressed HLA-A*6802 is mislocalized as an SS-uncleaved labile species that might be immediately targeted to the MG-132-sensitive degradation pathway.

To examine whether T7-tagging at the N-terminus of HLA-A*6802 might artificially affect its ER translocation efficiency, we expressed T7-tagged (T7-SS) and non-tagged (SS) species of HLA-A*6802 proteins (both of which possess Flag-tags following their SS) in HeLa cells. Microsomal fractions were prepared from these cells and were probed with the anti-Flag antibody. We found that the presence of the N-terminal T7 tag did not attenuate the incorporation of HLA species into the microsomal fraction compared with its N-terminally non-tagged counterpart (Fig. 1d, Flag-signal in 100,000 xg ppt. fraction). This result suggests that addition of the N-terminal T7 tag had little effect on the efficiency of ER translocation in this system.

### Signal sequence-uncleaved form of HLA-A is degraded by proteasomes

**Figure 2:**
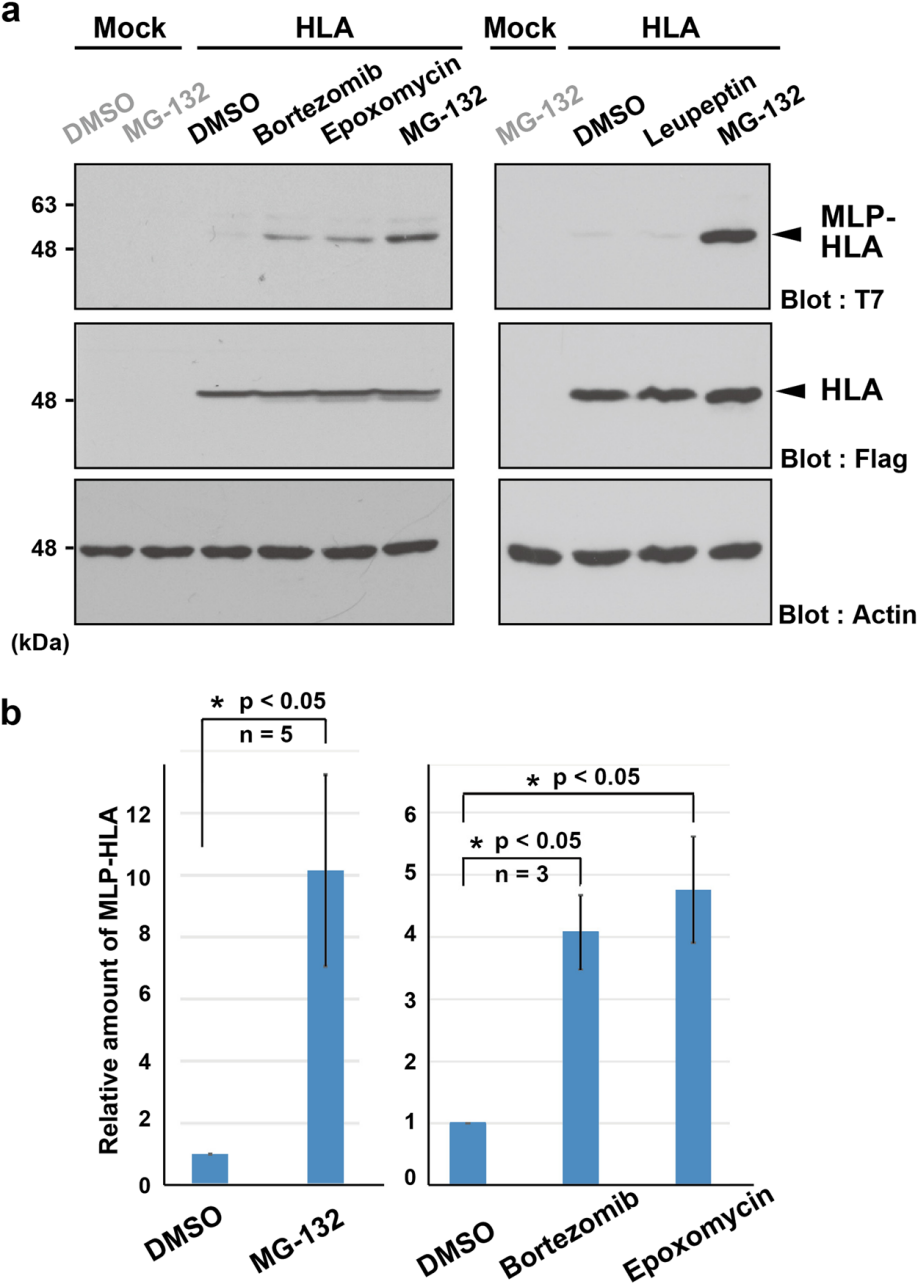
Proteasome is responsible for the degradation of the SS-uncleaved form of HLA-A. (**a**) T7-tagged mislocalized species of HLA-A*6802 protein (MLP-HLA) are degraded in a proteasome-dependent manner. HeLa cells expressing T7-tagged HLA-A*6802 were treated with 2 μM bortezomib, 10 μM epoxomicin, 10 μM Leupeptin and 10 μM MG-132. At 2 h after inhibitor treatments, the cells were lysed and the accumulation of HLA-A*6802 proteins was examined by either anti-T7 or anti-Flag antibodies. Mock indicates empty vector transfection. DMSO solvent was used as a negative control for inhibitor treatments. Actin was used as a loading control. (**b**) The graph quantitatively displays the data of the above blot signals as mean ± S.D. calculated from at least 3 independent experiments. *P < 0.05 compared with DMSO control. Full-length images of blots are presented in Supplementary Fig. S2.

MG-132 is a commonly used inhibitor for various intracellular proteases such as for proteasomes, cathepsin, and calpain. To identify the responsible protease for the rapid elimination of T7-tagged HLA-A*6802, we examined the effects of a series of protease inhibitors. Treatment of HeLa cells with bortezomib and epoxomicin, both of which inhibit proteasomes with higher specificity than MG-132 (although less effectively), also stimulated the accumulation of the T7-positive defective form of HLA-A*6802 (Fig. 2a,b), as was the case for MG-132 (Figs 1c and 2b). In contrast, treatment of HeLa cells with leupeptin (Ac-Leu-Leu-Arg-CHO), a structurally related lysosomal inhibitor, failed to block the accumulation of the T7-positive signal (Fig. 2a), excluding the possibility of the lysosomal degradation of this defective protein. These observations suggest a major contribution of the proteasome in the degradation of the SS-uncleaved form of HLA-A*6802.

### BAG6 is essential for the elimination of the signal sequence-uncleaved form of HLA-A

It has been reported that the mislocalized PrP protein is a client for the BAG6-mediated cytosolic degradation pathway^2,3^. We have also recently identified that the SS-defective form of the TMD protein IL-2Rα is a client of the UBQLN4/BAG6 complex^4^. Thus, we examined the possible participation of BAG6 in the elimination of the SS-uncleaved form of HLA-A protein.

**Figure 3:**
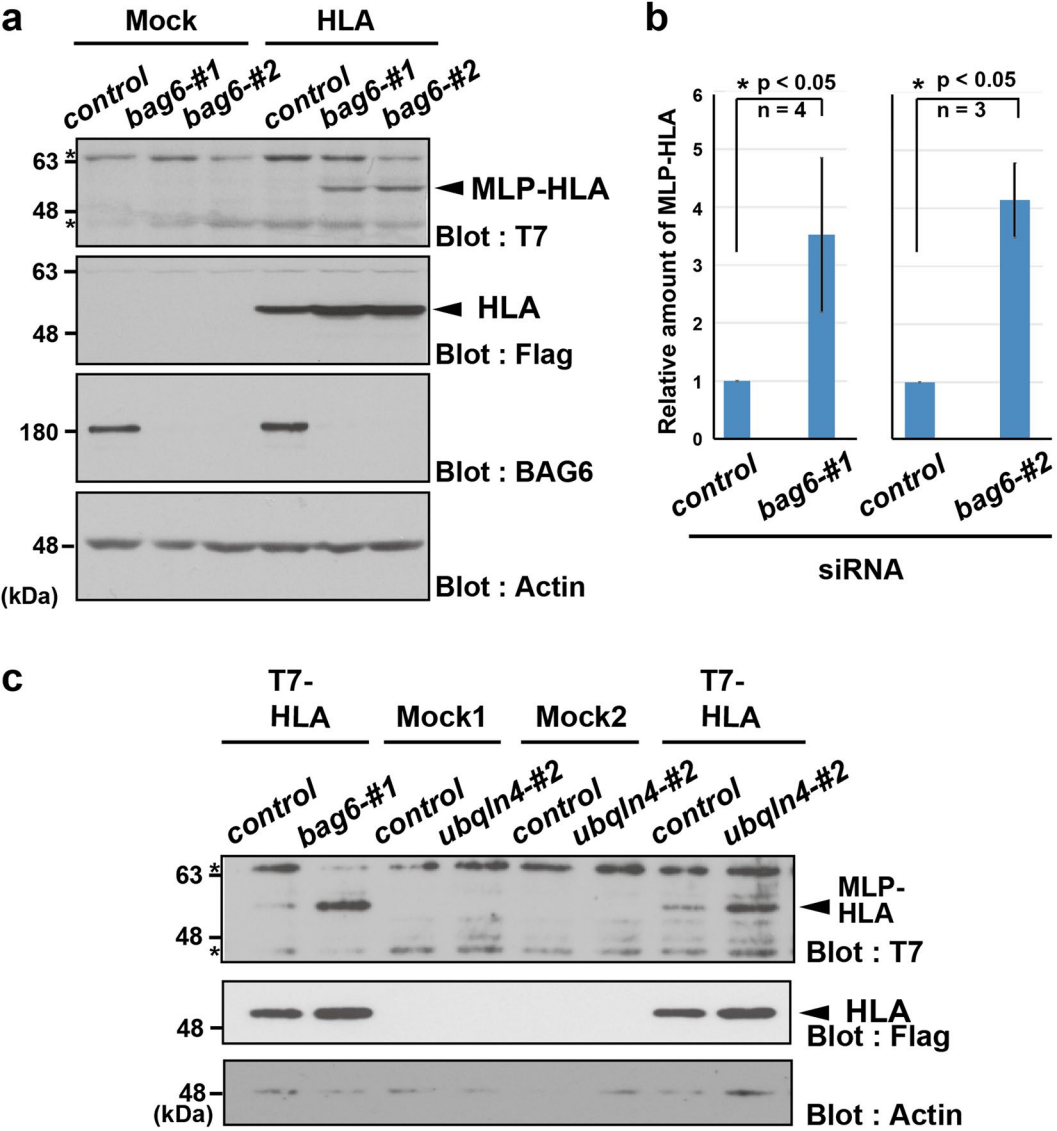
BAG6 is essential for the elimination of the SS-uncleaved form of HLA-A. (**a**) HeLa cells were transfected with two distinct siRNA duplexes for *bag6* (siRNA#1 and #2) or control siRNA (5 nM each). At 48 h after siRNA transfection, T7-tagged HLA-A*6802 was expressed in the cells. At 24 h after HLA-A*6802 transfection, the cells were harvested and lysates were probed with anti-T7- and anti-Flag-antibodies as in Fig. 2a. The band indicated by an asterisk is non-specific. The BAG6 blot indicates the efficient depletion of BAG6 protein by its siRNA. Actin was used as a loading control. (**b**) The graph indicates the quantified data of the anti-T7 signals in the above blot as mean ± S.D. calculated from at least 3 independent experiments. n = 4 for *bag6* siRNA#1. n = 3 cells for *bag6* siRNA#2. *P < 0.05 compared with control siRNA cells. (**c**) HeLa cells were transfected with siRNA duplexes for *bag6* (siRNA#1), *ubqln4* (siRNA #2) or control siRNA as in (**a**). Full-length images of blots are presented in Supplementary Fig. S3.

To estimate the impact of BAG6 on the elimination of mislocalized HLA-A*6802, we compared the levels of the T7-positive signal in the presence or absence of *bag6* small interfering RNA (siRNA). As shown in Fig. 3a, immunosignals for the T7-positive form of HLA-A*6802 were barely detected under the control siRNA condition, whereas the massive accumulation of the Flag-tagged mature form of HLA-A*6802 was obvious in the same cell lysates. Importantly, we found that *bag6* knockdown induced the large accumulation of T7-positive (and thus SS-cleavage defective) HLA-A*6802 polypeptide, even in the absence of proteasome inhibitors (Fig. 3a), whereas the Flag-tagged signal was scarcely affected by *bag6* siRNA. To avoid possible off-target effects of the double-stranded RNA used, we designed two independent siRNA constructs for *bag6* and verified whether knockdown experiments with these two independent double-stranded RNAs provided identical results. As shown in Fig. 3b, both constructs significantly stimulated the accumulation of T7-positive SS-uncleaved HLA-A*6802, compared with when a universal negative control siRNA construct was used, showing that defective HLA-A protein with an uncleaved SS can indeed be stabilized in BAG6-suppressed cells. In addition, knockdown of UBQLN4, a BAG6-associated UBA-domain protein^4^, also stimulated the accumulation of T7-positive SS-uncleaved HLA-A*6802 (Fig. 3c).

### BAG6 is essential for the solubilization of mislocalized HLA-A

**Figure 4:**
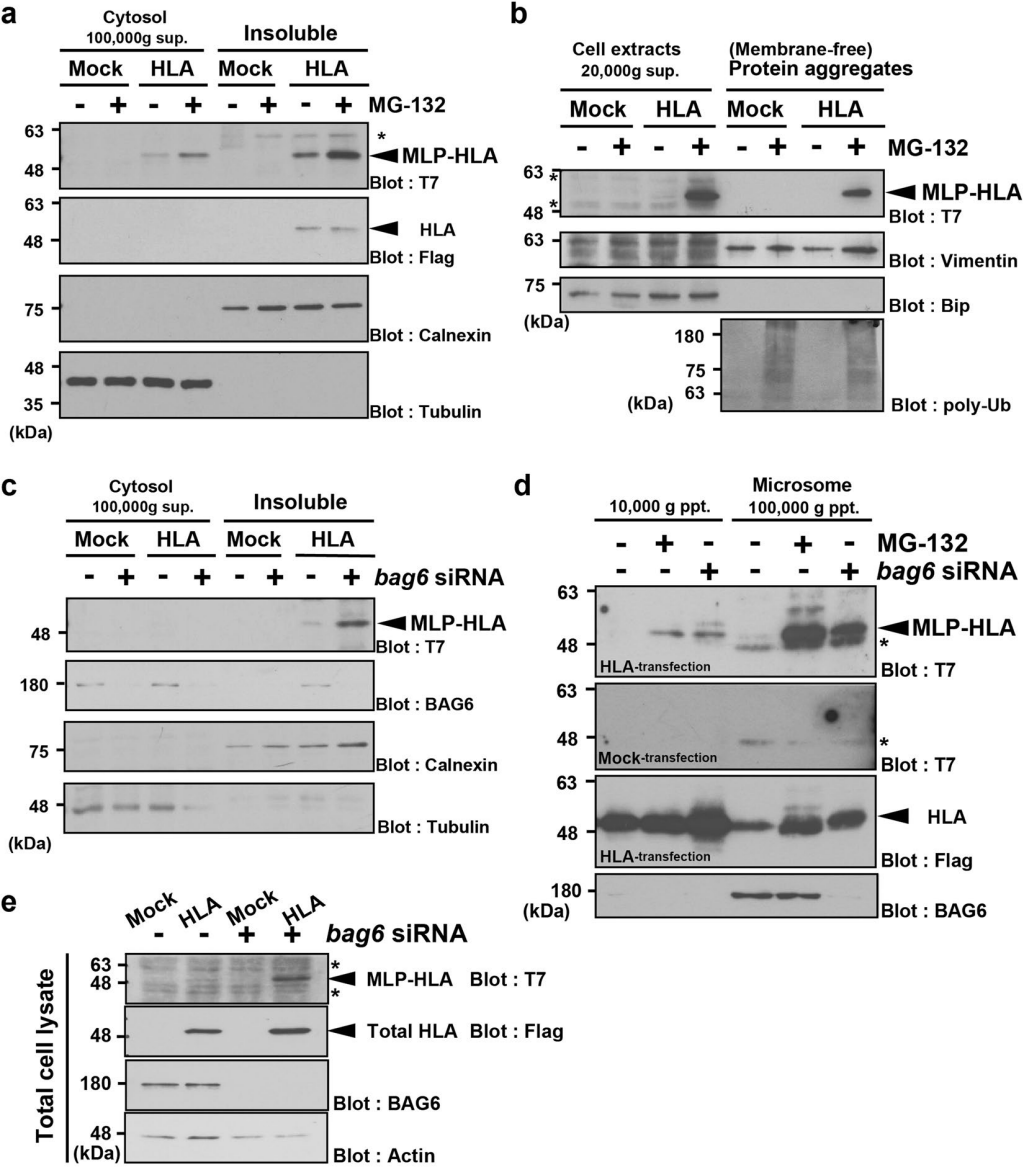
BAG6 depletion stimulates the accumulation of mislocalized HLA-A protein in the membrane-associated fraction. (**a**) HeLa cells expressing N-terminally T7-tagged HLA-A*6802 were treated with (+) or without (−) MG-132 for 4 h, and were fractionated into cytosolic and membrane-associated/insoluble fractions. Both T7- and Flag-immunosignals were detected. Tubulin was used as a cytoplasmic marker and calnexin was used for the ER membrane fraction. (**b**) HeLa cells with an N-terminally T7-tagged HLA-A*6802 expression vector (or a mock vector) were treated with (+) or without (−) 10 μM MG-132, and harvested with PBS containing 1% Triton X-100 detergent. The detergent-insoluble protein fractions were subjected to anti-T7 western blot analysis. The successful isolation of microsome-free protein aggregates was verified by anti-Bip (negative), anti-Vimentin (positive) and anti-polyubiquitin FK2 (positive) immunoblots. (**c**) HeLa cells transfected with 5 nM *bag6* siRNA#1 (+) or control siRNA (−) were fractionated into cytosolic and insoluble fractions, and were probed with anti-T7-antibodies to detect the MLP form of HLA-A*6802. The BAG6 blot indicates the depletion of BAG6 protein by its siRNA. (**d**) HeLa cells were transfected with an expression vector encoding N-terminally T7-tagged HLA-A*6802 protein with a Flag-tag at downstream of the SS (HLA-transfection). Empty vector transfection was used as a negative control (Mock transfection). The cells were treated with 10 μM MG-132 (for 4 h) or *bag6* siRNA (for 72 h) as indicated. Microsome fractions were prepared from these cells, and they were probed with anti-T7 and anti-Flag antibodies. The microsome fraction contained Flag-positive HLA signals irrespective of the presence or absence of MG-132, while the T7-positive HLA signal was absent in this membrane fraction without MG-132. Note that *bag6* siRNA greatly stimulated the accumulation of T7-positive MLP-HLA in the microsomal fraction. (**e**) HeLa cells were transfected with *bag6* siRNA#1 duplex or control siRNA (5 nM each), and the lysates were probed with anti-T7- or anti-Flag-antibodies to detect MLP or total HLA-A*6802 species in non-fractionated cell extracts, respectively. The BAG6 blot indicates the near complete depletion of BAG6 protein by its siRNA. Actin was used as a loading control. Asterisks indicate non-specific signals. Full-length images of blots are presented in Supplementary Figs S4 and S5.

Cell fractionation experiments revealed that most of the Flag-positive signals of HLA-A*6802 can be detected in the insoluble fraction (Fig. 4a), and that the signal was not stabilized by treatment with MG-132 (Fig. 4a), suggesting that successfully ER-assembled HLA-A*6802 is quite stable. In contrast, the T7-positive signals were sensitive to MG-132, and could be detected in both the soluble and insoluble fractions (Fig. 4a). We suggest that most of the mislocalized HLA-A*6802 species with an unprocessed SS are eliminated by proteasome-dependent pathways before reaching the lumen of the ER, either in the cytoplasm or on the cytoplasmic face of the ER membrane. Because the fractionation protocol used in Fig. 4a did not discriminate between the ER membrane and insoluble protein aggregates, we performed additional experiments to determine whether HLA accumulates in the aggregates or in the microsome fraction. We isolated detergent-insoluble protein aggregates from MG-132-treated cells, and probed these with the anti-T7-tag antibody. As shown in Fig. 4b, the Bip-negative/polyubiquitin-positive insoluble fraction indeed contained the T7 signal, although most of the T7-signal remained in the Bip-positive supernatant fraction. These observations suggest that only a portion of MLP-HLA co-precipitated with protein aggregates.

Similar to the case of MG-132 treatment, *bag6*-knockdown resulted in large accumulation of T7-positive HLA-A*6802 protein in the insoluble fraction (Fig. 4c), as in the case of the total cell lysates (Fig. 4e). In contrast to the case of MG-132, we failed to detect the T7 signal in the soluble fraction of *bag6*-knockdown cells (Fig. 4c). We next examined whether the microsomal membrane fraction contains MLP-HLA. We found that *bag6* siRNA (as well as MG-132 treatment) greatly stimulated the accumulation of T7-positive MLP-HLA in the microsomal fraction (Fig. 4d). Conversely, the microsome-free cytosolic fraction (100,000 × g supernatant) did not contain the T7 signal (Fig. 4c). These results suggest that the majority of MLP-HLA is localized in the microsomal membrane fractions when BAG6-mediated degradation was blocked. Since signal peptidase is known to cleave the SS immediately after ER-luminal translocation, T7-positive HLA protein should not be inserted into the ER lumen. Thus, it is likely that the SS-uncleaved HLA protein might be attached to the cytosolic surface of the ER membrane, a platform for the rapid degradation of cytosolic substrates (such as CL1) with exposed hydrophobicity. These results suggest that the SS-uncleaved form of HLA-A*6802 protein is solubilized through BAG6, and that such a solubilization process might be necessary for the targeted proteasomal degradation of mislocalized HLA-A*6802 protein.

### Cellular distribution of SS-uncleaved HLA-A under BAG6 suppression

**Figure 5:**
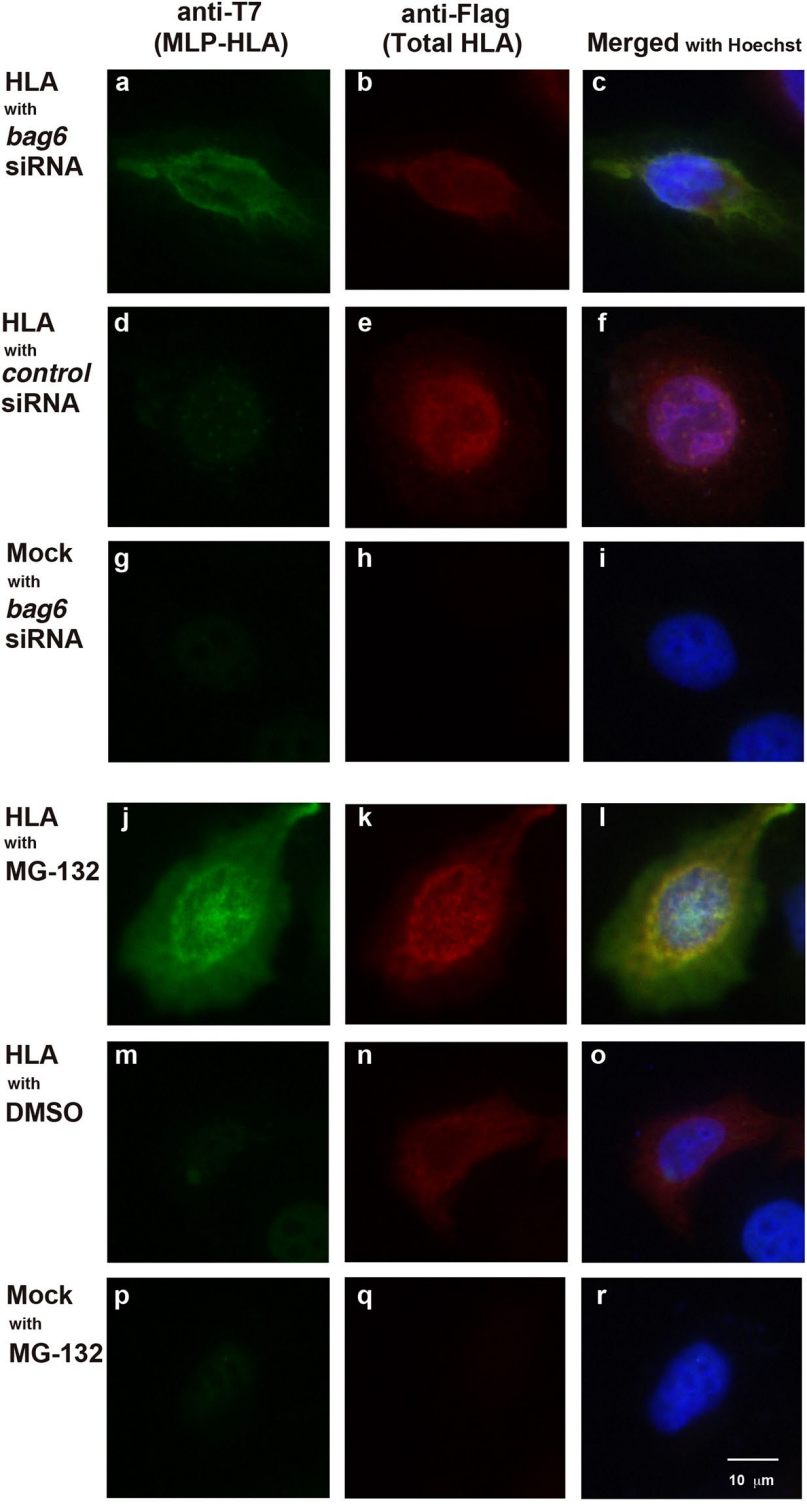
Immunocytochemical detection of the SS-uncleaved form of defective HLA-A. HeLa cells were transfected with an expression vector encoding N-terminally T7-tagged HLA-A*6802 (with Flag-tag downstream of the SS), and probed with the anti-T7 antibody to examine the cellular distribution of the SS-uncleaved form of HLA-A. Total HLA species were detected using the anti-Flag antibody as red signals, while SS-uncleaved MLP-HLA species were stained using the anti-T7 antibody as green signals. Nuclei were stained with Hoechst 33342 as blue, and merged with the anti-T7 and anti-Flag immunosignals. Before harvesting the cells, they were treated with or without *bag6* siRNA or MG-132, respectively, as indicated. Note that all photographs of the T7 and Flag immunosignals, respectively, were taken under identical exposure times throughout the experiments. Scale bar, 10 μm.

To examine the cellular distribution of the mislocalized form of HLA-A, we performed immunocytochemical analysis. As shown in Fig. 5, total HLA signals (Flag-immunosignals, shown in red) were detected around the ER irrespective of the presence or absence of MG-132 or *bag6* siRNA treatment. Conversely, MLP-HLA signals (T7-immunosignals, shown in green) were detected only in the presence of MG-132 or *bag6* siRNA (compare Fig. 5a,d,j,m). These observations further support the notion that the SS-uncleaved form of HLA-A is eliminated by the BAG6- and proteasome-mediated degradation systems. Please note that the cytoplasmic T7 signals observed in MG-132-treated cells could barely be detected in *bag6* knockdown cells (compare Fig. 5a,b,j,k), in accordance with the observation in Fig. 4c. In addition, microscopic observation failed to detect any sign of cytoplasmic HLA aggregates (Fig. 5a,j), even under MG-132 treatment, although cell fractionation analysis suggested a portion of T7-HLA was detected in the protein aggregate fraction (Fig. 4b). This may be due to its relatively small population compared with membrane-associated MLP-HLA.

### BAG6 is essential for the elimination of artificially mislocalized HLA-A

**Figure 6:**
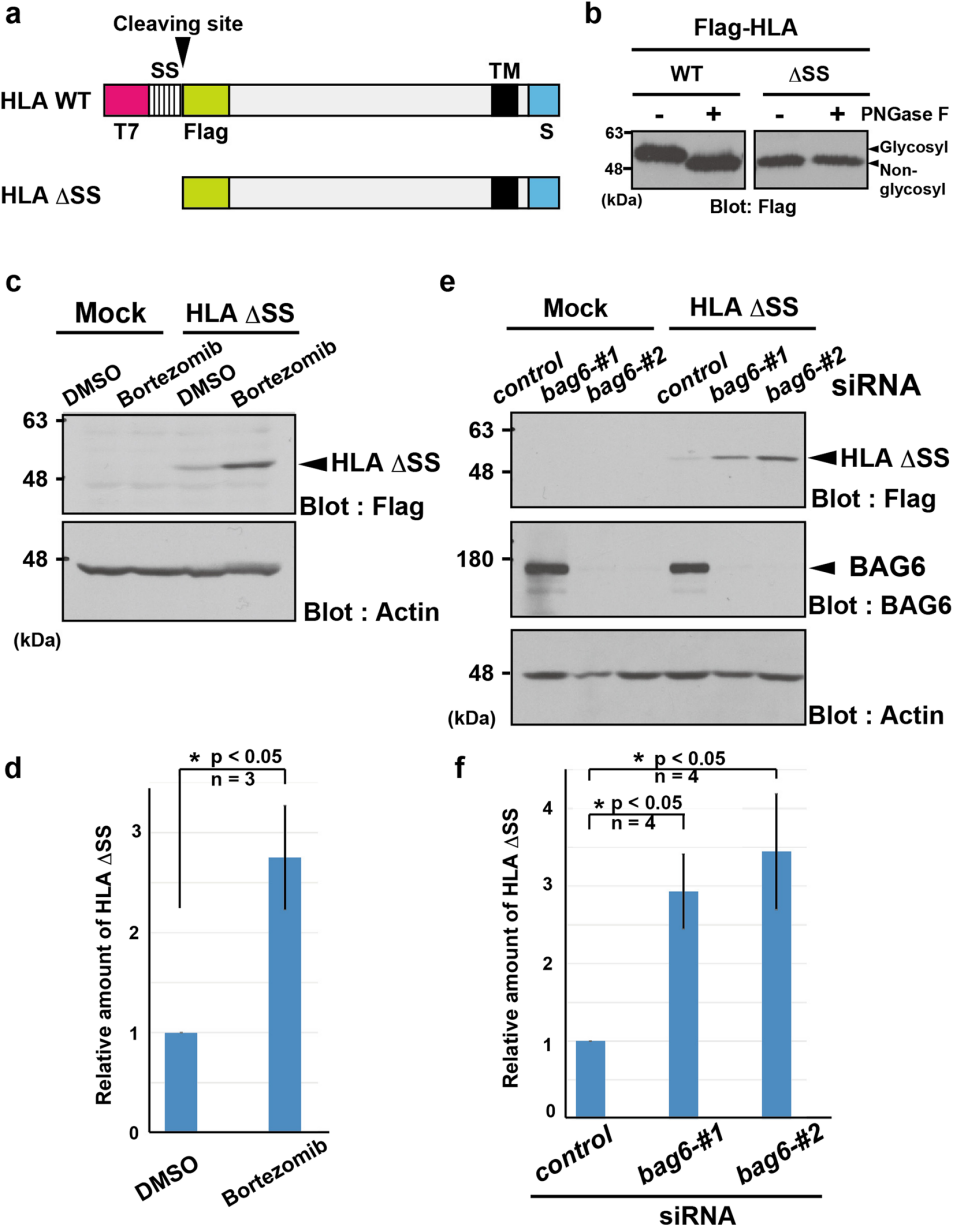
BAG6 is critical for the degradation of HLA-A ΔSS. (**a**) Schematic representation of the HLA-A*6802 deletion mutant used in this study. WT: wild-type, ΔSS: signal sequence-deleted mutant. (**b**) The band of Flag-HLA-A*6802 WT was shifted down by treatment with PNGase F, a deglycosylation enzyme, suggesting that this protein is glycosylated. However, glycosyl modification was barely detected for the Flag-HLA-A*6802 ΔSS, indicating that this mutant is not inserted properly into the ER membrane. (**c, d**) Mislocalized HLA-A ΔSS protein was stabilized by proteasome inhibitor. Flag-HLA-A ΔSS was expressed in HeLa cells and the cells were treated with (+) or without (−) 20 μM bortezomib for 4 h before harvesting. After western blotting with the indicated antibodies, anti-Flag immuno-signals were quantified. The data represent as mean ± S.D. calculated from 3 independent experiments. Asterisks indicate P < 0.05. (**e**,**f**) BAG6 is essential for degradation of the mislocalized HLA-A ΔSS. HeLa cells were transfected with two distinct siRNA duplexes for BAG6 (*bag6* siRNA#1 and #2) or control siRNA (5 nM each). At 48 h after siRNA transfection, Flag-tagged HLA ΔSS was expressed in the cells. At 24 h after Flag-HLA ΔSS transfection, the cells were harvested. After western blotting with the indicated antibodies, anti-Flag immuno-signals were quantified. The data represent as mean ± S.D. calculated from 4 independent experiments. Asterisks indicate P < 0.05. Actin was used as a loading control. Full-length images of blots are presented in Supplementary Fig. S6.

It has been reported that truncating the SS from the transmembrane receptor protein IL-2Rα results in the defective assembly of the polypeptide into the ER and its destabilization for cytoplasmic destruction^4,47^. To examine whether a SS-truncated variant of HLA-A*6802 protein could also serve as a model for MLPs, we prepared such a truncated derivative, HLA ΔSS (Fig. 6a). Note that the “ΔSS” form of HLA-A*6802 we prepared here and “SS-cleaved” HLA-A*6802 observed in previous experiments are conceptually distinct; “SS-cleaved” HLA-A is generated only after its ER luminal translocation and is thus localized in the ER (and other membrane fractions) as a mature form, while “HLA ΔSS” is considered not to be recognized by the SRP before ER translocation, and is localized in the cytoplasm (or cytoplasmic face of the ER) as a defective form. We confirmed that glycosyl modification of HLA ΔSS was not detected (Fig. 6b) and that it is stabilized by a proteasome inhibitor (Fig. 6c,d), in contrast to the case of ER-assembled (SS-cleaved) HLA-A*6802 (Fig. 2a, Flag blot).

Because BAG6 is critical for the degradation of IL-2Rα ΔSS^4^, we examined the possible participation of BAG6 in the metabolism of misassembled HLA ΔSS. We found that the suppression of endogenous *bag6* using siRNA greatly stimulated the accumulation of HLA ΔSS (Fig. 6e,f). This observation supports the idea that BAG6 is essential for the degradation of the mislocalized form of HLA-A*6802 protein.

**Figure 7:**
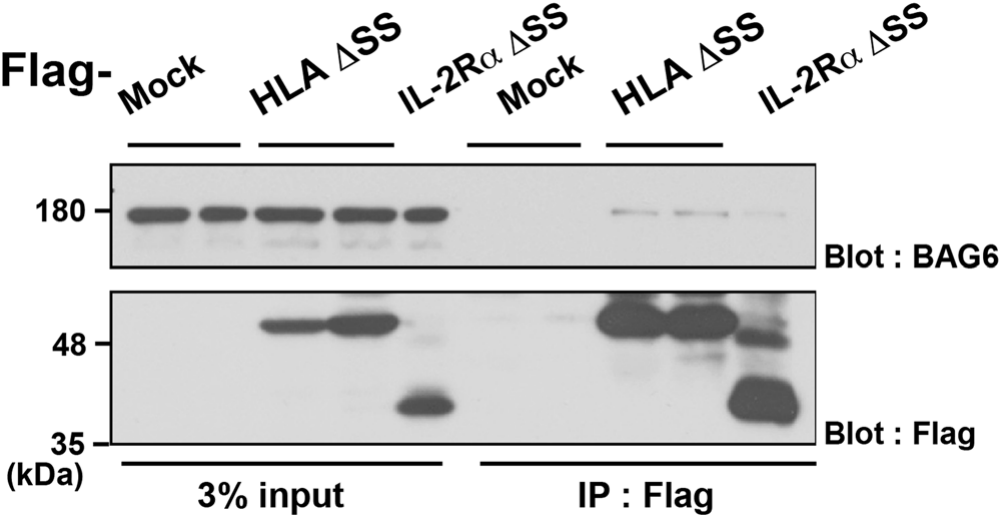
BAG6 recognizes mis-assembled HLA-A. Flag-tagged HLA ΔSS was immunoprecipitated from HeLa cell lysates, and its co‐precipitation with endogenous BAG6 was analyzed with an anti-BAG6 antibody. Mock indicates empty vector transfection. BAG6 co-precipitation with Flag-IL-2Rα ΔSS was used as a positive control. Full-length images of blots are presented in Supplementary Fig. S6.

BAG6 is known to associate with newly synthesized defective proteins^19,24^. To reveal how BAG6 regulates the turnover of misassembled HLA-A*6802 protein, we examined its physical interaction with HLA ΔSS. We immunoprecipitated Flag-tagged HLA ΔSS from HeLa cell lysates and examined its interaction with endogenous BAG6. We found that mislocalized HLA-A*6802 protein co-precipitated with BAG6, though tiny amount, as with IL-2Rα ΔSS^4^ (Fig. 7). These observations support the idea that BAG6 preferentially recognizes and contributes to the quality control of misassembled HLA proteins.

## Discussion

Recent progress in genome analysis has suggested that nearly one-fifth of polypeptides encoded in the genome are putative membrane proteins^48–50^. Accordingly, the aberrant production of mislocalized membrane proteins, even at a low percentage for an individual gene product, may have a non-negligible impact on cellular homeostasis as a whole. However, the detection of mislocalized TMD proteins is usually difficult, as such defective species are thought to be removed immediately upon synthesis in the cytosol. In this study, we attempted to assess one such cryptic portion of a defective TMD protein, HLA-A*6802, as a representative example, via T7-tagging upstream of its SS (Fig. 1a). The results showed that we were able to discriminate mislocalized species of HLA-A*6802 from its successfully assembled counterparts, and succeeded in showing that the proteasome is indeed indispensable for the cytosolic degradation of the SS-uncleaved form of defective TMD protein (Fig. 2). It may be possible that our use of the transient expression of HLA-A*6802 via a plasmid may overload the endogenous SRP machinery and increase the possibility of its mislocalization. It is true that difficulties with SRP recognition might occur when the ratio of the SRP to its client substrates is overwhelmed^4^. However, considering the fact that more than several thousand endogenous membrane/secretory proteins are produced physiologically via the SRP pathway within a mammalian cell^48–50^, and HLA-A*6802 is a one of such an SRP clients, the over-production of this protein by several fold should not overload the endogenous SRP machinery, although we do not have direct evidence to support this idea at the present time.

HLA-ΔSS, another model BAG6 substrate used in this study, is never recognized by the SRP, and thus, this model mutant protein is different from the accidentally mislocalized wild-type HLA product. However, our previous study showed that knockdown of the SRP increased the mislocalization of TMD proteins and their association with UBQLN4^4^, suggesting that mislocalization might occur when SRP recognition is impaired. In this sense, HLA-ΔSS works as an appropriate model to study the quality control system for such defective TMD proteins that fail to be recognized by the SRP.

Mislocalized proteins are selectively degraded by the proteasome^34^. This type of cytoplasmic, non-ERAD degradation machinery for ER proteins has been defined as a preemptive quality control system, which can be fine-tuned to reduce the efficiency of translocation during ER stress in an SS-sequence-specific manner^6–8,51^. It has been reported that a cell endogenous MHC class I-restricted peptide, derived mainly from proteasomal degradation products of newly synthesized defective polypeptides^52–56^, contains the cleavage site of the ER signal peptidase^57^. Because SS peptides targeted for ER insertion are cleaved off immediately during their co-translational assembly into the lumen of the ER^40^, this uncleaved epitope peptide could be produced prior to translocation into the ER^57^. In the present study, we showed that a part of HLA-A*6802 protein can be mislocalized with uncleaved SS, and suppression of BAG6 greatly strengthened the T7-positive signals of such a defective species (Figs 3 and 4). In agreement with this observation, we recently identified that the mislocalized form of the single-pass TMD protein IL-2Rα is a client of the UBQLN4/BAG6 complex under the specific condition of SS-/SRP-deficiency^4^. It was reported that ablating the SS of a type I TMD protein results in its default delivery to the cytosol, accelerated turnover, and enhanced peptide presentation for MHC class I molecules^47,58^. Indeed, IL-2Rα is mistargeted to the cytosol due to a lack of SRP engagement, resulting in the rapid degradation of nascent proteins and thus serving as an opportunity to provide an epitope peptide efficiently^47^. Although there might be redundant pathway(s) for the role of BAG6 in antigen presentation^59^, our results support the hypothesis that BAG6 might act as a critical factor for the proteasome-mediated degradation of a mislocalized TMD protein that possesses a non-cleaved SS (with a T7-tag) at its N-terminus. It might be interesting to note that HLA is one the most polymorphic self-gene products encoded in the human genome. As the degradation products of newly synthesized defective proteins are known to be a major source of antigenic peptides for MHC class I molecules (which are HLA proteins themselves), the degradation products derived from highly polymorphic HLA proteins might be the most suitable source of individual-specific self-antigens.

We still do not know what determines the specificity of BAG6, with so many candidate client proteins including hundreds of TA proteins^25,26^, thousands of mislocalized membrane proteins^2,4^, and millions of intrinsic newly synthesized defective translational products^13,19^. There might be some preference for their specific targeting to the degradation machinery, which remains to be determined. Because the HLA-restricted anti-tumor epitope peptide PSCA (amino acids 14-22) contains a cleavage site for the ER signal peptidase^57^, and because PSCA protein contains a highly hydrophobic stretch at its C-terminus, as in other known BAG6-preferring clients, an interesting challenge for the future will be to determine whether mislocalized SS-uncleaved membrane protein species might be general clients for the BAG6-mediated protein quality control machinery. Clarification of the linkage between defects in the metabolism of BAG6 clients and various human diseases linked to tumor etiology, neurodegenerations, and defective immune responses will be a relevant prospect for extensive future investigations.

## Materials and Methods

### Plasmid construction

Full-length cDNAs of HLA-A*6802 (GenBank accession numbers: U03861.1) was amplified by PCR from cDNA libraries derived from HeLa cells. The PCR fragments were cloned into pCI-neo-based mammalian expression vectors (Promega) with N-terminal 3xT7-, internal 3xFlag-, and C-terminal S-tags with their products. Vectors were used for experiments after verification of the sequence of inserted DNA.

### Mammalian cell culture and transfection

HeLa cells were cultured in Dulbecco’s modified Eagle’s medium (Wako) supplemented with 10% heat-inactivated calf serum at 37 °C under 5% CO_2_ atmosphere. Transfections of the expression vectors were perfomed with HilyMax (Dojindo, Japan) or polyethylenimine “MAX” transfection reagent (Polysciences, Inc.) according to the protocols supplied by the manufacturers. At 24 hr after transfection, the cells were harvested and subjected to immunological analysis unless otherwise noted. All experiments were performed in accordance with ethical guidelines in Tokyo Metropolitan University and the licensing committee approving this experiment.

### RNA interference

*bag6* and *ubqln4* depletion was performed as described previously^4,19^ with independent duplex siRNA sequences of;

5′-UUUCUCCAAGAGCAGUUUAtt-3′ (*bag6* siRNA#1),

5′-CAGAAUGGGUCCCUAUUAUtt-3′ (*bag6* siRNA#2),

5′- CUCAAUAACCCUGAACUCAtt-3′ (*ubqln4* siRNA#2).

In addition, MISSION siRNA Universal Negative Control 1 (SIGMA Aldrich) was used as a general negative control in every experiment. Transfections of HeLa cells with duplex siRNA were performed using Lipofectamine 2000 (Invitrogen) according to the protocol provided by the manufacturer. The efficacy of each siRNA was verified by immunoblot with anti-BAG6 antibody^19^.

### Immunological analysis

For western blot analyses, whole cell lysates or the immunoprecipitates were subjected to SDS-PAGE and transferred onto Polyvinylidene Fluoride transfer membrane (GE Healthcare, Pall Corporation). The membranes were then immunoblotted with specific antibodies as indicated and then incubated with horseradish peroxidase-conjugated antibody against mouse or rabbit immunoglobulin (GE Healthcare), followed by detection with ECL western blotting detection reagents (GE Healthcare), Clarity^TM^ Western ECL substrate (Bio-RAD) or Immobilon Western (Millipore).

For immunoprecipitation analysis, HeLa cells were washed with ice-cold phosphate-buffered saline (PBS) and lysed with immunoprecipitation (IP) buffer containing 20 mM Tris-HCl pH 7.5, 5 mM EDTA, 150 mM NaCl, 1% Nonidet P-40, 10 mM N-ethylmaleimide, 25 μM MG-132 and protease inhibitor cocktail (Nacalai tesque). The lysates were sonicated, centrifuged at 20,630 x g for 20 min at 4 °C, and mixed with 4~10 μL of anti-Flag M2 affinity gel (Sigma) for 10 min at 4 °C. After the beads had been washed five times with the IP buffer, the immuno-complexes were eluted by SDS sample buffer.

The following antibodies were used in this study: anti-T7-tag monoclonal (Novagen, 695223), anti-Flag M2 monoclonal (Sigma, F3165), anti-Flag polyclonal (Sigma, F7425), anti-S-tag (Santa Cruz Biotechnology, Santa Cruz, CA), anti-BAG6/Scythe^19^, anti-β-actin (Sigma), anti-Bip (BD), anti-Vimentin (Santa Cruz), anti-Calnexin (Sigma), anti-polyubiquitin FK2 (MBL, Nagoya, Japan), anti-Tubulin (Santa Cruz).

### Soluble and insoluble fractionation

The expression vectors of HLA-A*6802 with N-terminal T7-tag was transfected into HeLa cells. After 22 h of transfection, subcellular fractionation was performed using a EzSubcell Extract (ATTO) according to the protocol provided by manufacturer. Tubulin was used as a cytoplasmic marker, while calnexin was used for the ER membrane/insoluble fraction marker. Both the cytosolic and insoluble fractions were probed with anti-T7-antibody to detect MLP form of HLA-A*6802 (Fig. 4a,c).

### Microsome preparation from HeLa cell extracts

HeLa cells were harvested at 600 × *g* centrifugation and gently crushed using a 23 G needle with extraction buffer (50 mM HEPES, pH 7.4, 10 mM KCl, 1 mM DTT, 0.25 M sucrose, 200 μM PMSF) at 4 °C. The cell lysates were centrifuged at 1,000 × *g* for 5 min and subsequently separated at 10,000 × *g* for 20 min at 4 °C. The 10,000 × *g* supernatants were further centrifuged at 100,000 × *g* for 60 min at 4 °C. The resulting supernatants were used as cytosolic fractions, while the precipitates were used as microsomal fractions. After washing the microsomal fraction with the extraction buffer, the precipitates were dissolved in SDS-PAGE sample buffer for western blot analysis. The successful isolation of microsomal fractions was verified by anti-Calnexin or anti-Bip immunoblots.

### Preparation of the detergent-insoluble protein aggregate fraction

HeLa cells were treated with 10 μM MG-132 for 12 h, and harvested with PBS containing 1% Triton X-100. The cell lysates were centrifuged at 20,000 x*g* for 20 min at 4 °C, and the resulting precipitates were washed extensively with 1% Triton X-100 solution. The Triton X-100-insoluble protein aggregates were dissolved in SDS-PAGE sample buffer for western blot analysis. The successful isolation of microsome-free protein aggregates was verified by anti-Bip, anti-Vimentin and anti-polyubiquitin FK2 immunoblots.

### Microscopic observations

For immunocytochemical observations, HeLa cells were grown on micro coverglass (Matsunami, Japan), fixed by incubating in 4% paraformaldehyde for 30 min on ice, and permeabilized with 0.1% Triton X-100 for 3 min at room temperature. Fixed cells were blocked with 3% calf serum in PBS and reacted with anti-T7 monoclonal (mouse IgG) and anti-Flag polyclonal (rabbit IgG) antibodies at room temperature for 1 h. An Alexa^TM^ 488-conjugated anti-mouse IgG antibody and Alexa^TM^ 594-conjugated anti-rabbit IgG antibody (Molecular Probe) were used as secondary antibodies at 1:1000 dilution. To observe nuclei, the cells were treated with 2.5 µg/mL Hoechst 33342. Immunofluorescent images were obtained with BIOREVO BZ9000 fluorescence microscope (Keyence, Japan).

### Data availability

The data analyzed during the current study are available from the corresponding author on request.

## Electronic supplementary material

Supplementary information
