# Supplementary material for: Elimination of a signal sequence-uncleaved form of defective HLA protein through BAG6
Source: Sci Rep. 2017 Nov 6;7:14545. doi: 10.1038/s41598-017-14975-9 (PMC5674028; doi:10.1038/s41598-017-14975-9)
Supplement: Supplementary file 1 — Supplementary information [file 41598_2017_14975_MOESM1_ESM.pdf]

Supplementary information

Elimination of a signal-sequence uncleaved form of defective HLA protein through BAG6

Koki Yamamoto, Mizuki Hayashishita, Setsuya Minami, Kanji Suzuki,

Takumi Hagiwara, Aya Noguchi and Hiroyuki Kawahara

Laboratory of Cell Biology and Biochemistry, Department of Biological Sciences,

Tokyo Metropolitan University, Tokyo 192-0397, Japan

Original blot for Figure 1b

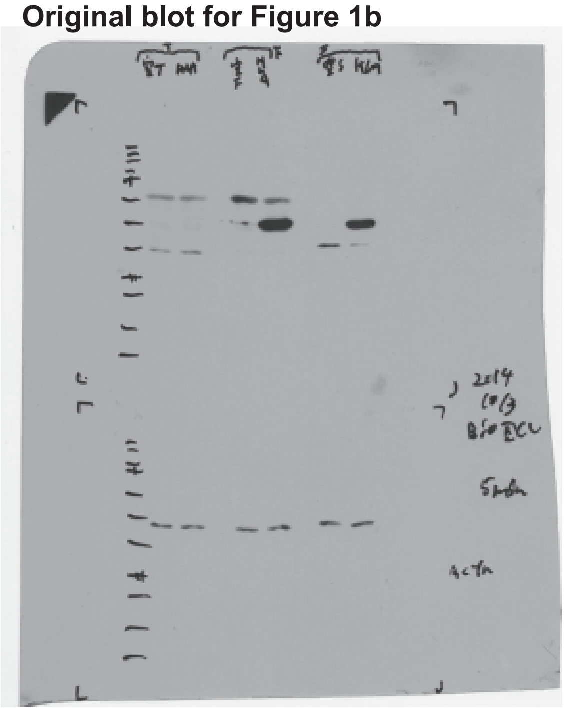

Original blot for Figure 1c

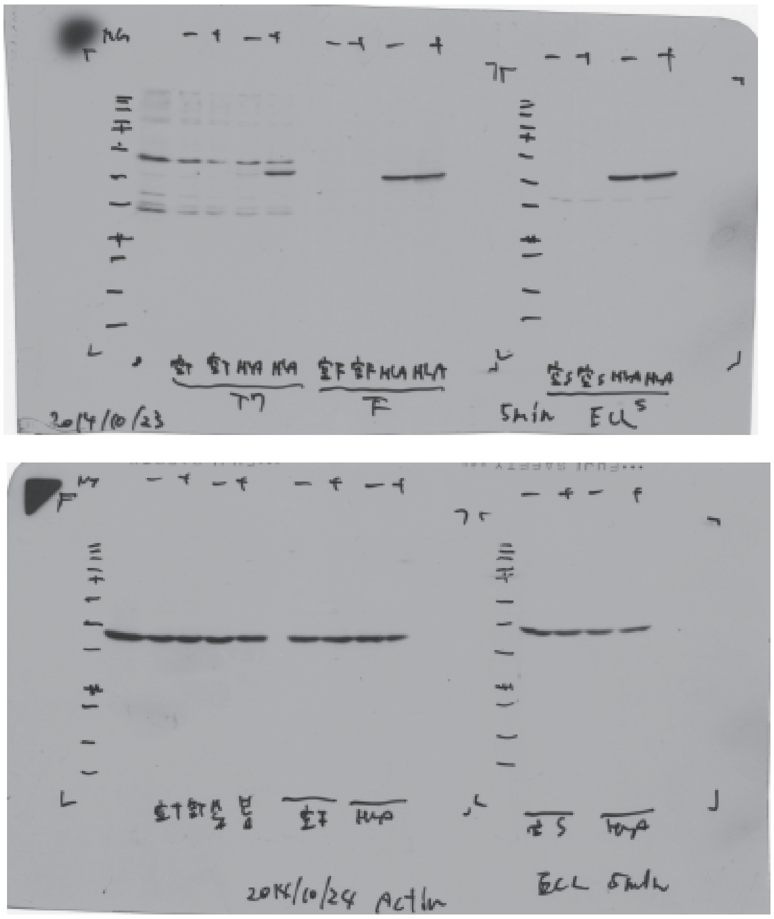

Original blot for Figure 1d

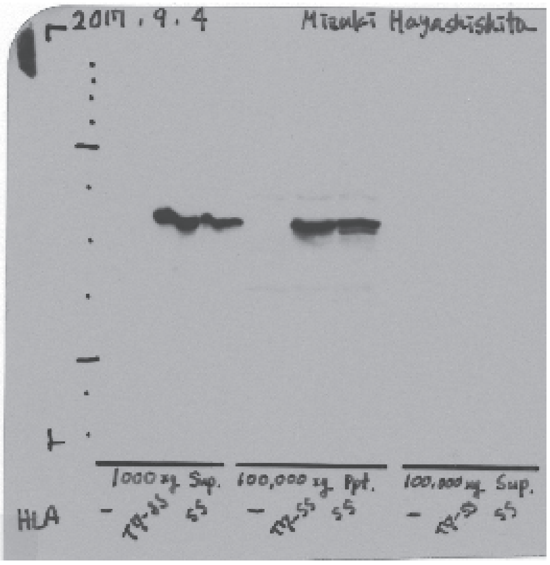

Supplementary Figure S1, Yamamoto et al.

Original blot for Figure 2a

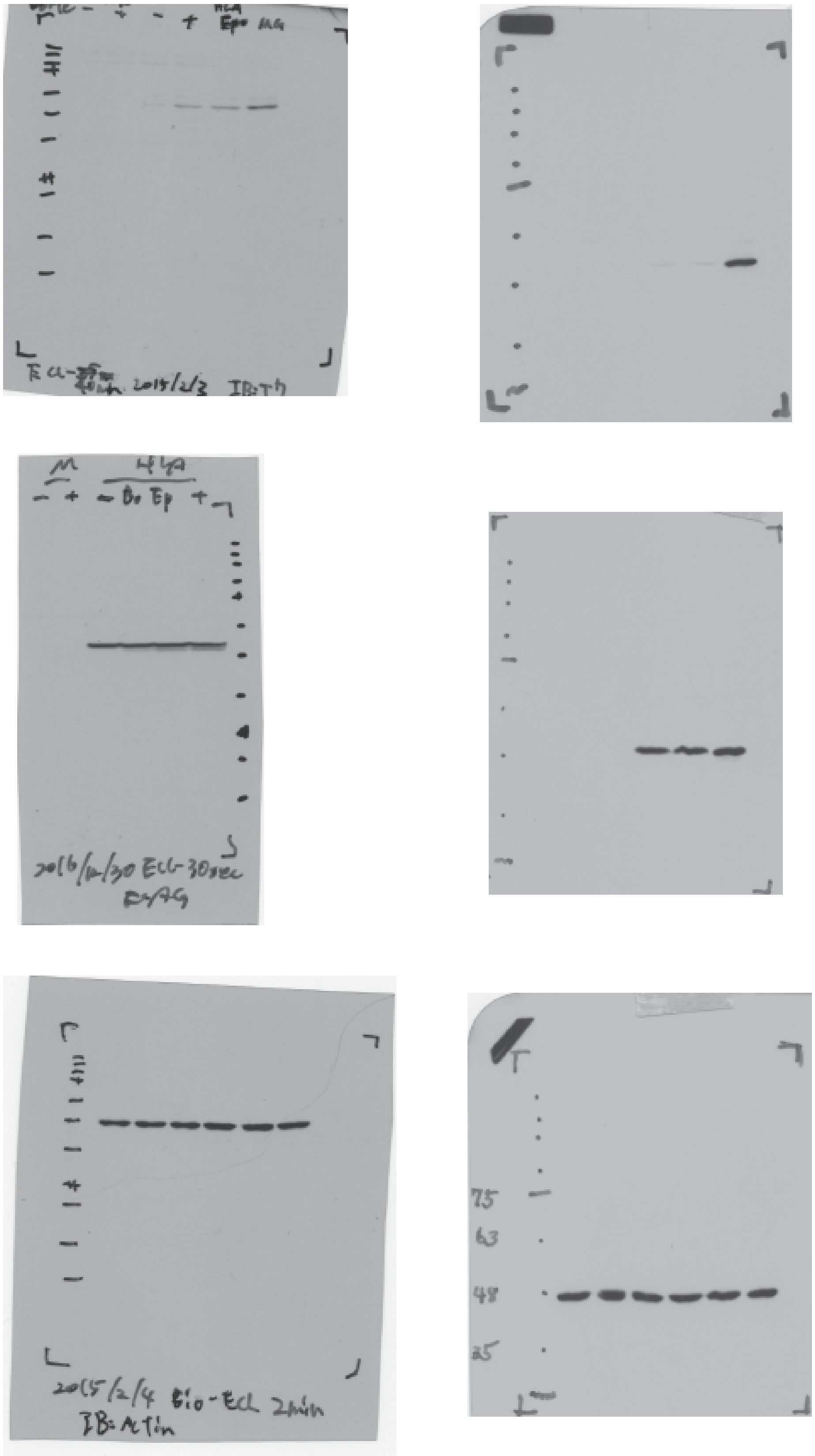

Supplementary Figure S2, Yamamoto et al.

Original blot for Figure 3a

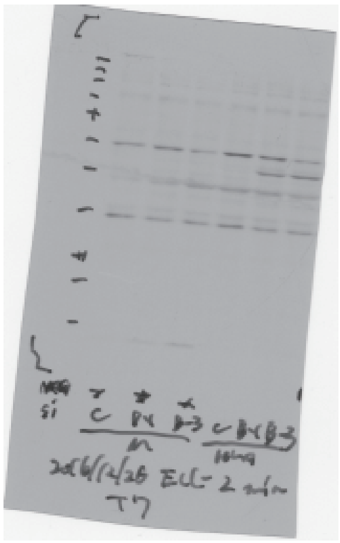

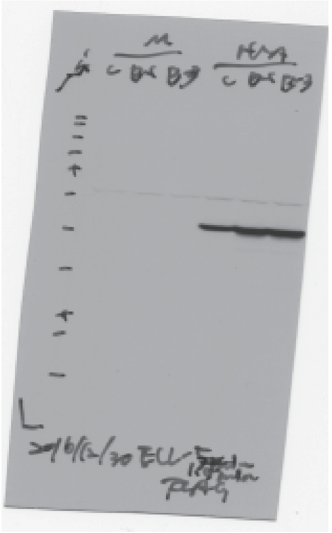

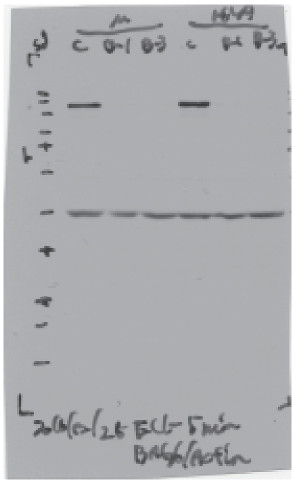

Original blot for Figure 3c

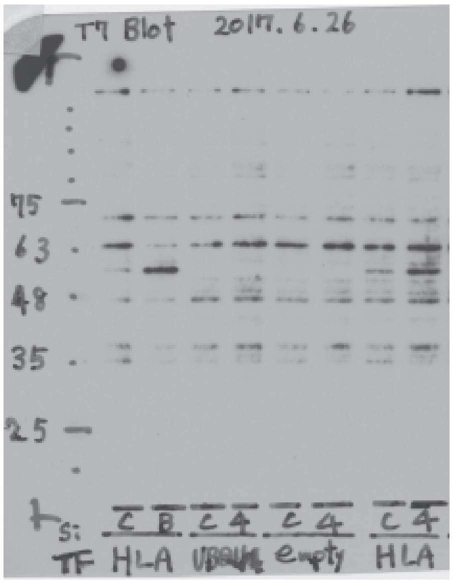

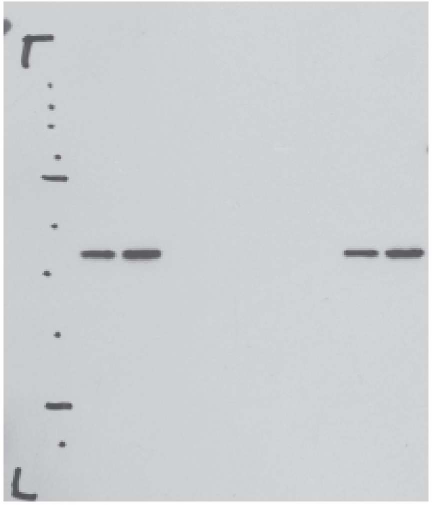

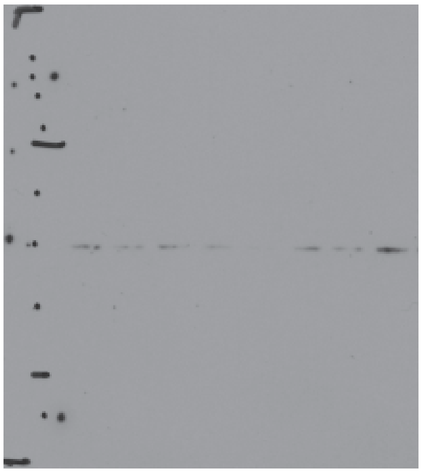

Supplementary Figure S3, Yamamoto et al.

Original blot for Figure 4a

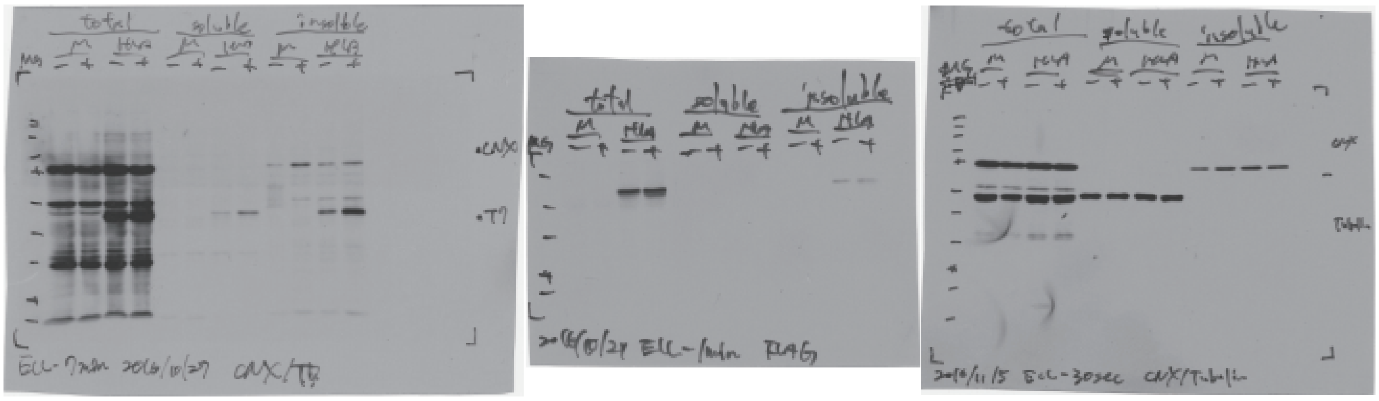

Original blot for Figure 4c

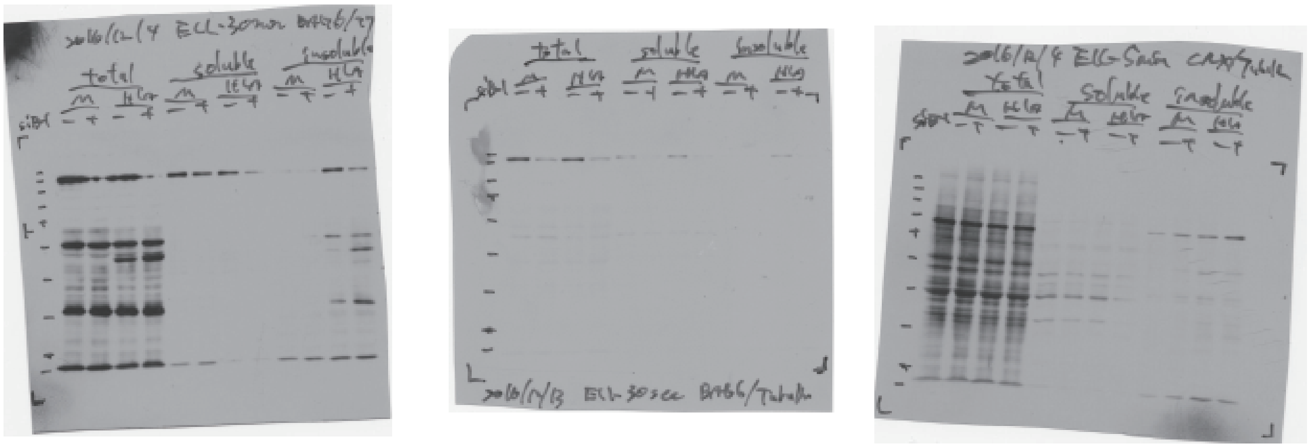

Original blot for Figure 4e

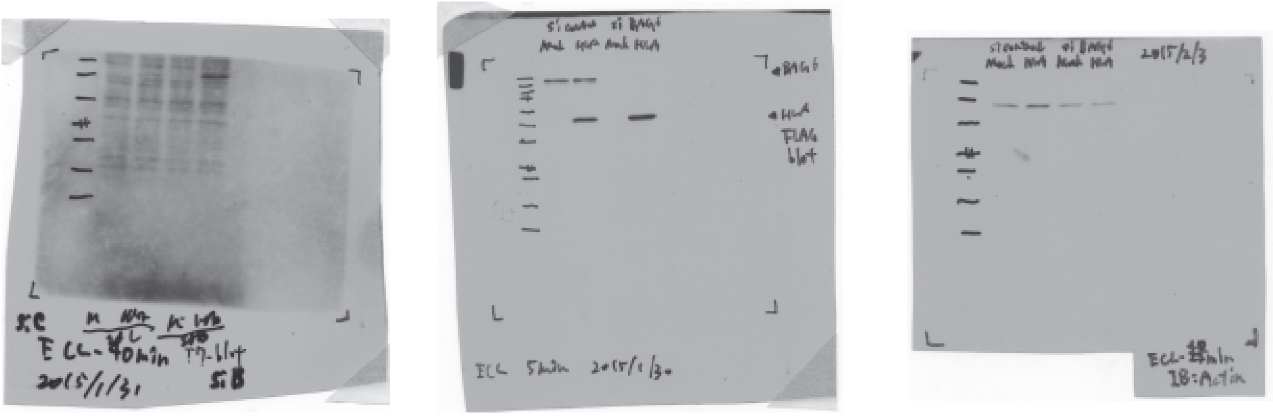

Supplementary Figure S4, Yamamoto et al.

Original blot for Figure 4b

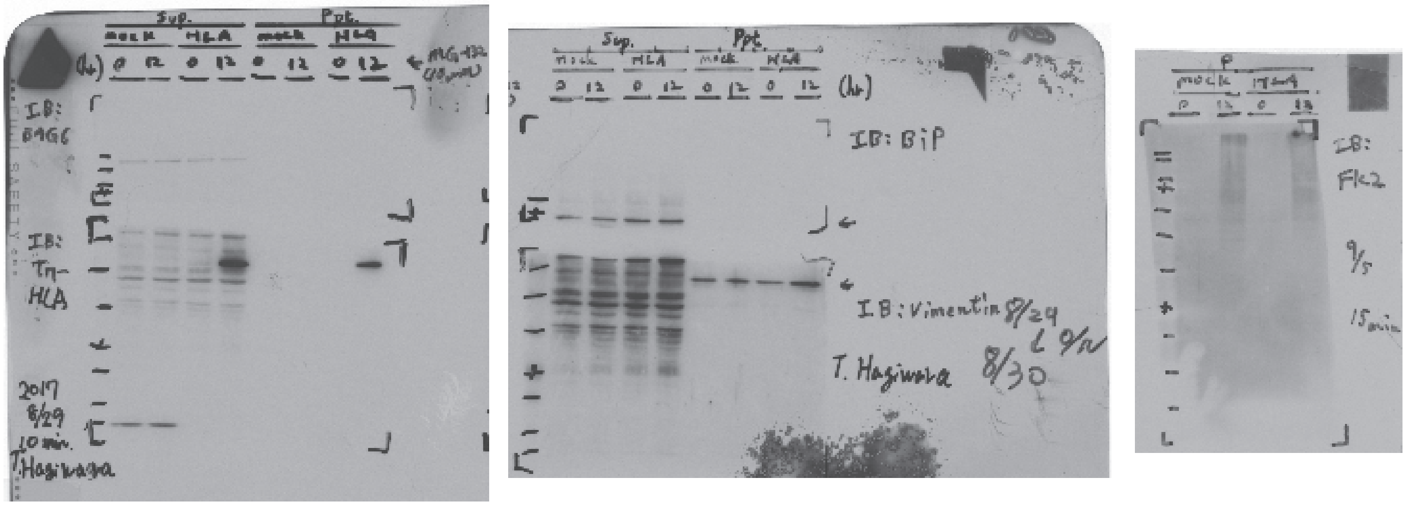

Original blot for Figure 4d

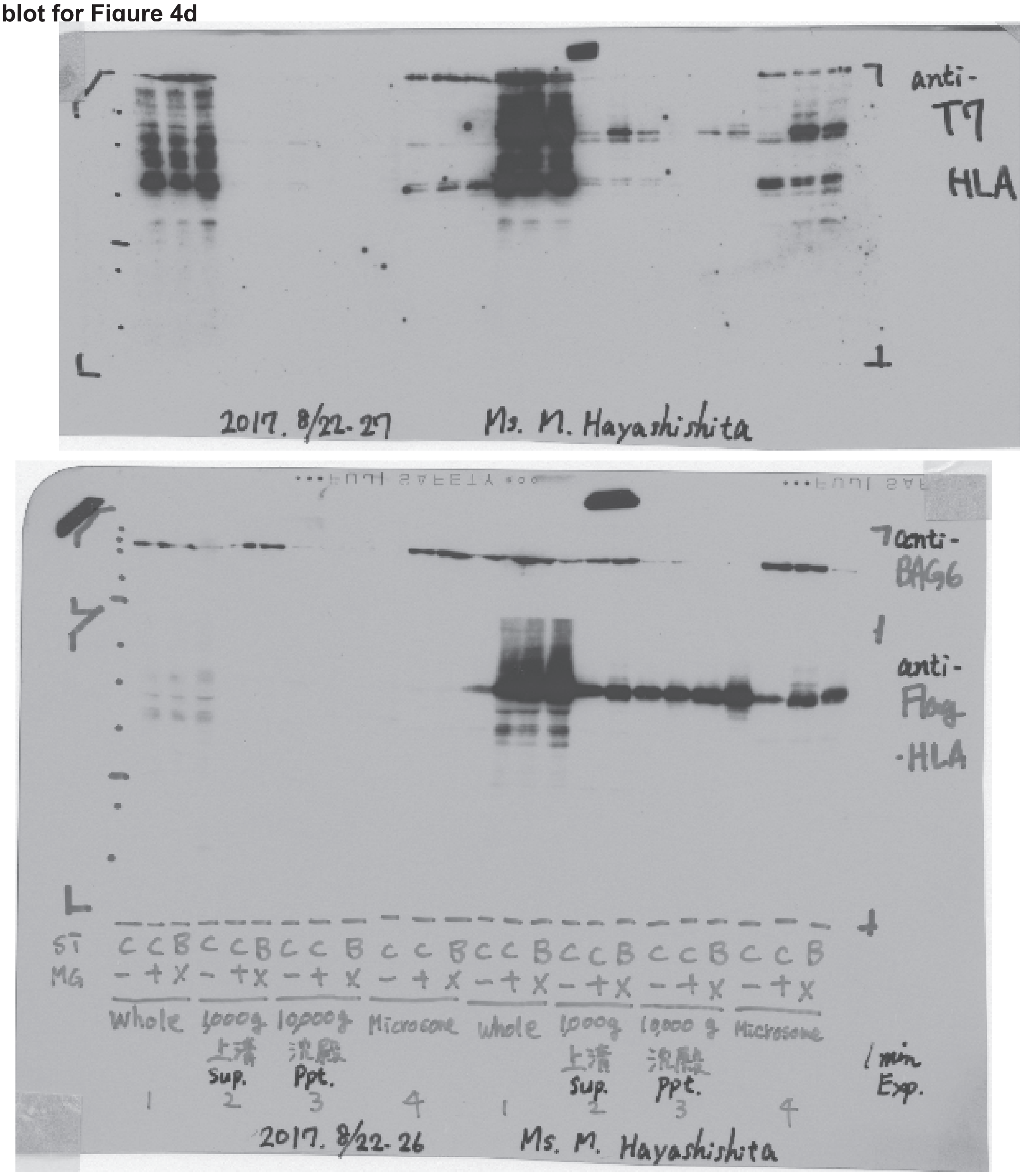

Supplementary Figure S5, Yamamoto et al.

Original blot for Figure 6b

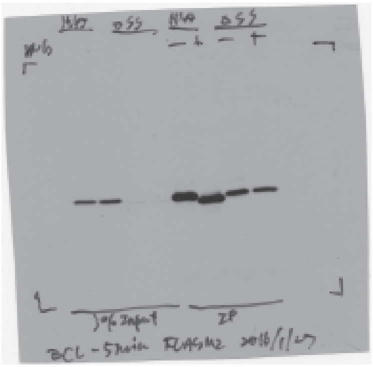

Original blot for Figure 6c

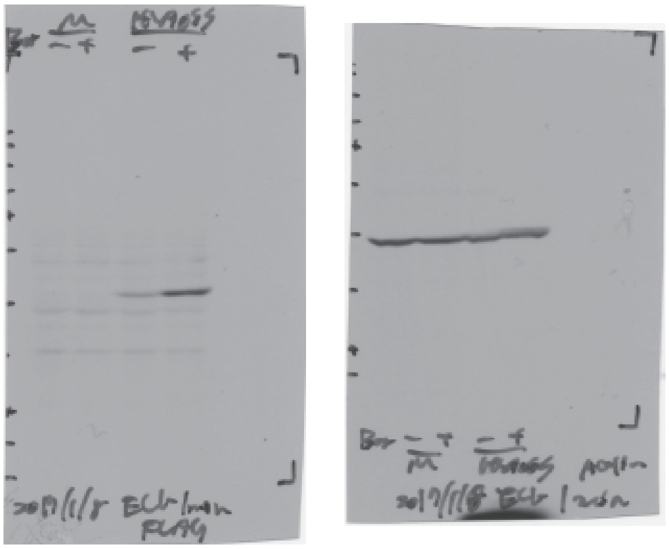

Original blot for Figure 6e

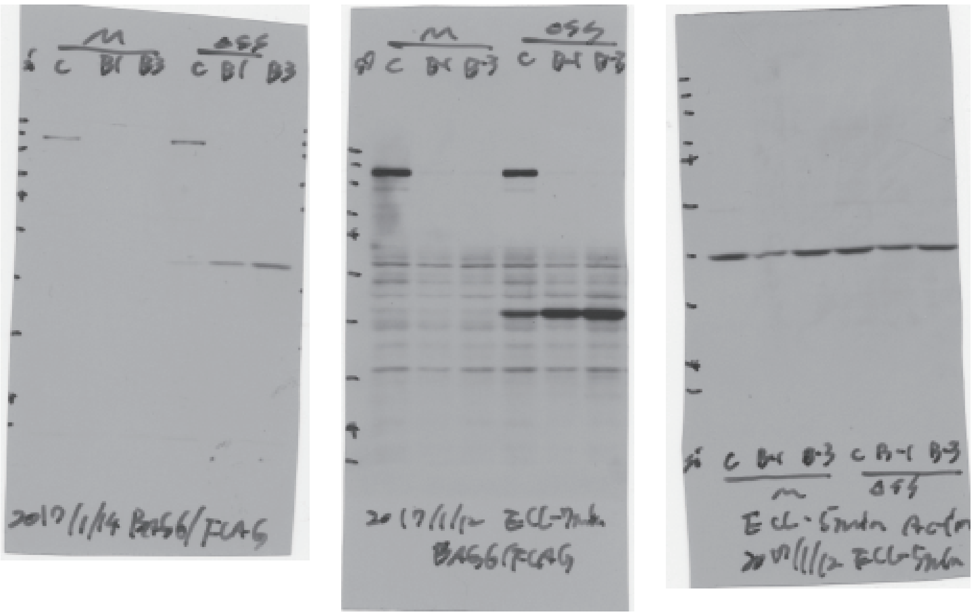

Original blot for Figure 7

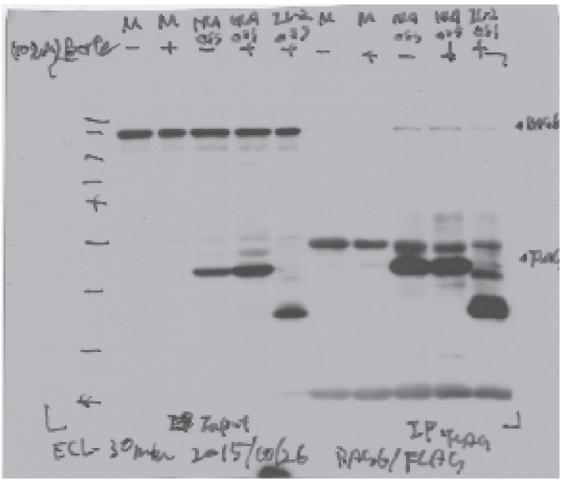

Supplementary Figure 6, Yamamoto et al.
